# Supplementary material for: Pd-Based Nano-Catalysts Promote Biomass Lignin Conversion into Value-Added Chemicals
Source: Materials (Basel). 2023 Jul 24;16(14):5198. doi: 10.3390/ma16145198 (PMC10384994; doi:10.3390/ma16145198)
Supplement: Supplementary file 1 [file materials-16-05198-s001.zip › materials-2501497-supplementary.pdf]

Supporting Materials

Pd-based nano-catalysts promote biomass lignin conversion into value-added chemicals

Ming Zhao [1,*], Liang Zhao [2,3,*], Xiao-Yan Zhao [2], Jing-Pei Cao [2], and Koh-ichi Maruyama [1]

[1] Department of Materials and Biology, National Institute of Technology, Akita College, Akita, 011-8511, Japan; maruko@akita-nct.ac.jp

[2] Jiangsu Province Engineering Research Center of Fine Utilization of Carbon Resources, China University of Mining & Technology, Xuzhou 221116, China; zhaoxiaoyan@cumt.edu.cn (X.-Y.Z.); caojingpei@cumt.edu.cn (J.-P.C.)

[3] Institute of Innovative Research, Tokyo Institute of Technology, 4259 Nagatsuta, Midori-ku, Yokohama 226-8503, Japan

[*] Correspondence: mingzhao@akita-nct.ac.jp (M.Z.); zhao.l.ae@m.titech.ac.jp (L.Z.)

Chemicals

Palladium (II) acetylacetonate [$Pd(acac)_2$, 99%], nickel (II) acetylacetonate [$Ni(acac)_2$, 99%], triphenylphosphine (TPP, 99%), tetrabutylammonium bromide (TBAB, 99%), trioctylphosphine oxide (TOPO, 98%), and oleylamine (OLA, 70%) were purchased from Sigma-Aldrich. 10 wt% Pd/C was purchased from Beijing Hawk Science & Technology Co. Ltd. All the reagents were used without further purification.

Table S1. Analysis of porous structure of the catalysts.

Sample	S_{BET} (m^2/g)[a]	V_{total} (m^3/g)[b]	D_{ave} (nm)[c]
Pd-Ni-P/C	134	1.16	3.52
Pd-Ni/C	96	0.55	3.15
Pd-P/C	90	0.70	3.19
Pd/C	834	0.51	3.16

[a] Measured by N_2 adsorption; [b] Calculated by BET method; [c] Measured by BJH method.

Table S2. Pd contents in different catalysts determined by ICP-MS.

Catalyst	Pd (wt%)
Pd/C	10
Pd-P/C	10.4

Pd-Ni/C	10
Pd-Ni-P/C	7.3

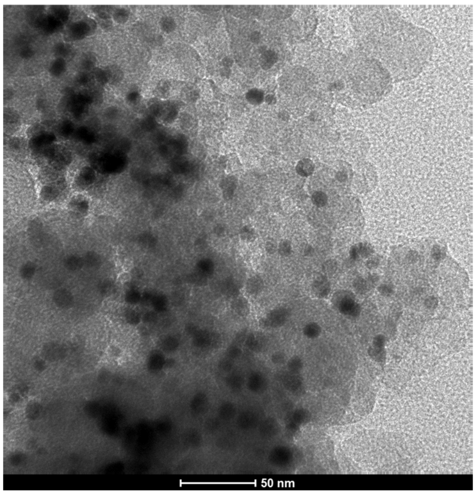

Figure S1. TEM image of commercial Pd/C.

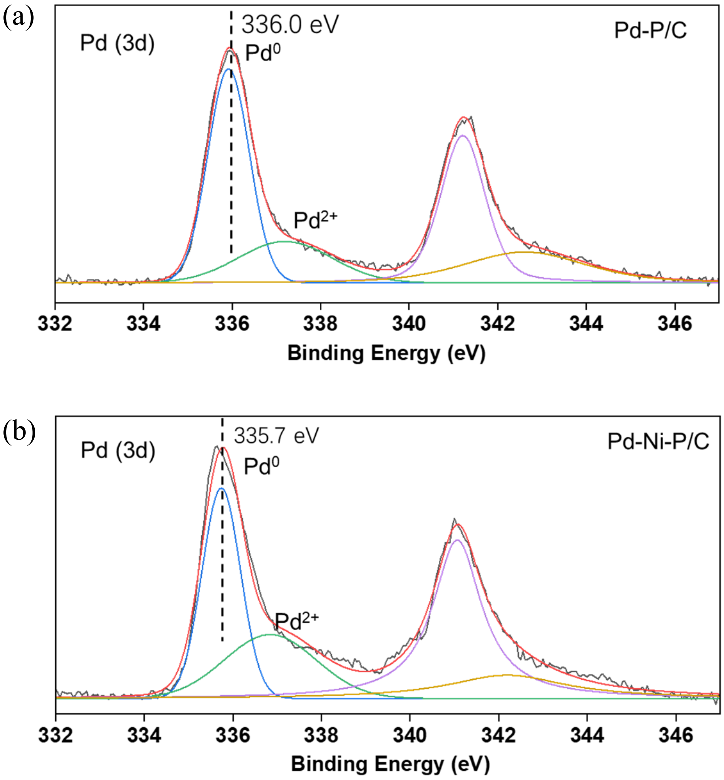

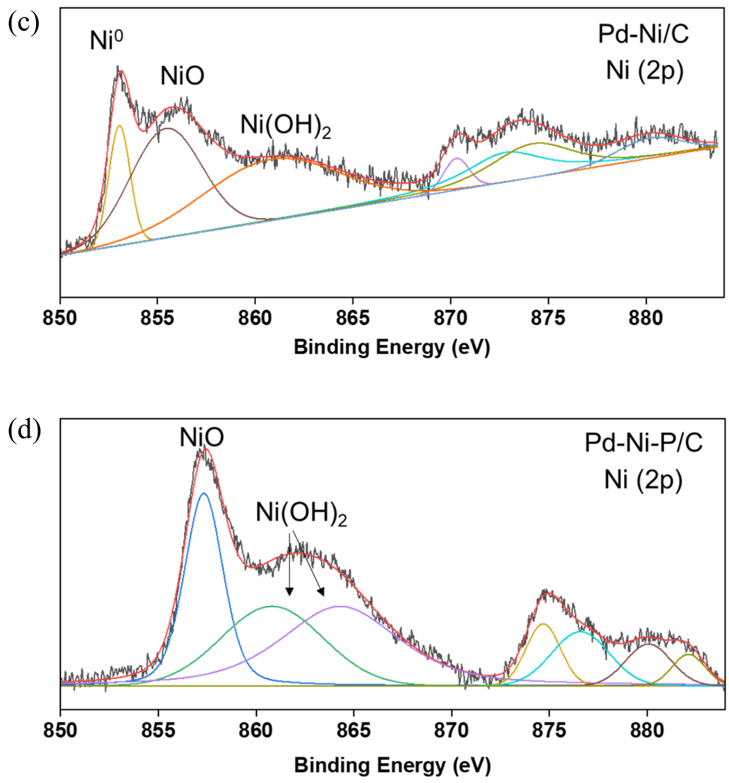

Figure S2. XPS spectra of different samples with electronic state analysis of Pd (a and b) and Ni (c and d) elements.

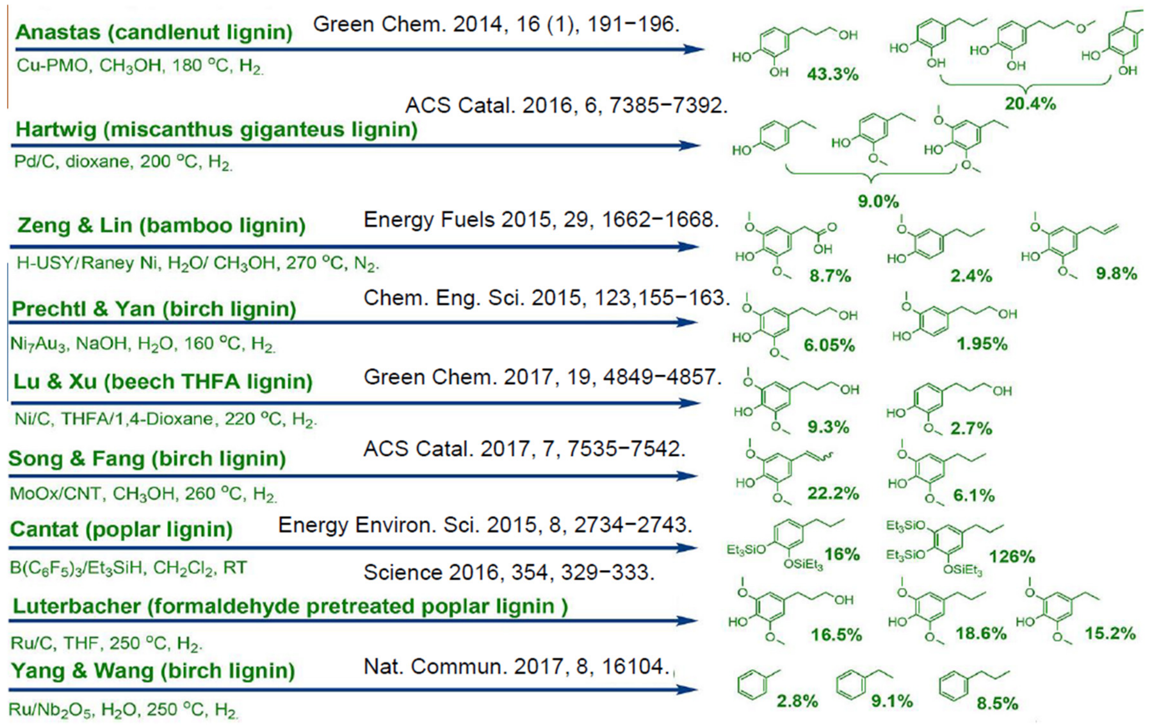

Figure S3. Product information of lignin depolymerization promoted by various previously reported metal-based catalysts.
